# Dietary index for gut microbiota and hypertension risk: a cross-sectional NHANES study

**DOI:** 10.3389/fnut.2025.1622058

**Published:** 2025-10-07

**Authors:** Xiaona Che, Xinqi Li, Lin Na, Yunfei Sun, Ziang Kong, Wenjing Cui, Jing Chang, Xin Xue

**Affiliations:** ^1^Department of Cardiology, The Second Hospital of Jilin University, Changchun, China; ^2^Department of Cardiology, Xi’an International Medical Center Hospital, Xi’an, China; ^3^Clinical Laboratory, The Second Hospital of Jilin University, Changchun, China

**Keywords:** dietary index, gut microbiota, gut microbiota dietary index, hypertension, National Health and Nutrition Examination Survey

## Abstract

**Background:**

Gut microbiota’s role in hypertension is emerging, but systematic studies on microbiota-linked dietary indices (DI-GM, BGMS, UGMS) remain limited.

**Methods:**

This study leveraged data from the National Health and Nutrition Examination Survey (NHANES) database spanning 1999–2020. A cross-sectional study design was employed to gather baseline information from 41,193 adult participants aged 20 years and older, encompassing sociodemographic characteristics and health-related factors. To investigate the associations between DI-GM, BGMS, UGMS, and the prevalence of hypertension, weighted logistic regression models, restricted cubic spline (RCS) analysis with three knots (positioned at the 10, 50, and 90th percentiles of the independent variables), and subgroup analyses were performed.

**Results:**

The study findings demonstrate that both DI-GM and BGMS are significantly and inversely associated with the prevalence of hypertension. Specifically, each one-unit increase in DI-GM was linked to a 4% reduction in hypertension risk (OR = 0.96, 95% CI: 0.94–0.98, *p* < 0.001), and each one-unit increase in BGMS was associated with a 5% decrease in hypertension risk (OR = 0.95, 95% CI: 0.92–0.97, *p* < 0.001). Further RCS analysis demonstrated a linear relationship between DI-GM and BGMS with hypertension risk. Additionally, subgroup analyses stratified by age, gender, BMI, and diabetes status exhibited robust results (P for interaction >0.05).

**Conclusion:**

DI-GM and BGMS exhibit significant inverse associations with hypertension prevalence, with BGMS displaying a stronger protective effect. No significant relationship was identified between UGMS and hypertension.

## Introduction

Hypertension is one of the leading causes of death and disability globally, accounting for approximately 9.4 million deaths annually (1, 2). According to existing literature, in 2010, approximately 31.1% of the global adult population—equivalent to 1.39 billion individuals—was affected by hypertension, with the global prevalence of the disease continuing to rise steadily (3). Studies have demonstrated that dietary modifications can significantly reduce the incidence of hypertension, as the absorption and metabolism of nutrients are substantially influenced by the gut microbiota and its metabolites (4). Current evidence indicates that both the composition of the gut microbiota and its associated metabolites play a pivotal role in the initiation and progression of cardiovascular diseases (5–7). Consequently, the gut microbiome is increasingly recognized as a potential target for novel therapeutic strategies aimed at preventing and managing hypertension.

Diet represents a critical environmental determinant that significantly shapes the compositional profile of the gut microbiota (8). In nutritional epidemiology, dietary indices serve as essential tools for quantifying dietary patterns (9, 10). Among the most widely used indices are the Healthy Eating Index (HEI), the Alternative Healthy Eating Index (aHEI), the Mediterranean Diet Score (MDS), and the DASH Diet (Dietary Approaches to Stop Hypertension) (11). While these indices effectively evaluate the relationship between dietary quality and health outcomes, their correlations with gut microbiota *α*/*β* diversity metrics exhibit heterogeneity (12–14). Unlike traditional dietary evaluation indicators (such as HEI, DASH), the Dietary Index for Gut Microbiota (DI-GM) systematically assesses the regulatory effects of 14 food/nutrient categories (10 beneficial components and 4 restricted components), enabling quantitative analysis of the health status of the gut microbiota.

This study leverages data from the National Health and Nutrition Examination Survey (NHANES) spanning 1999–2020, employing a cross-sectional study design to investigate the association between the dietary index for the microbiota and its components and the risk of hypertension. The aim is to provide scientific evidence supporting precise nutritional interventions for hypertension management.

## Methods

### Data source

**Figure 1:**
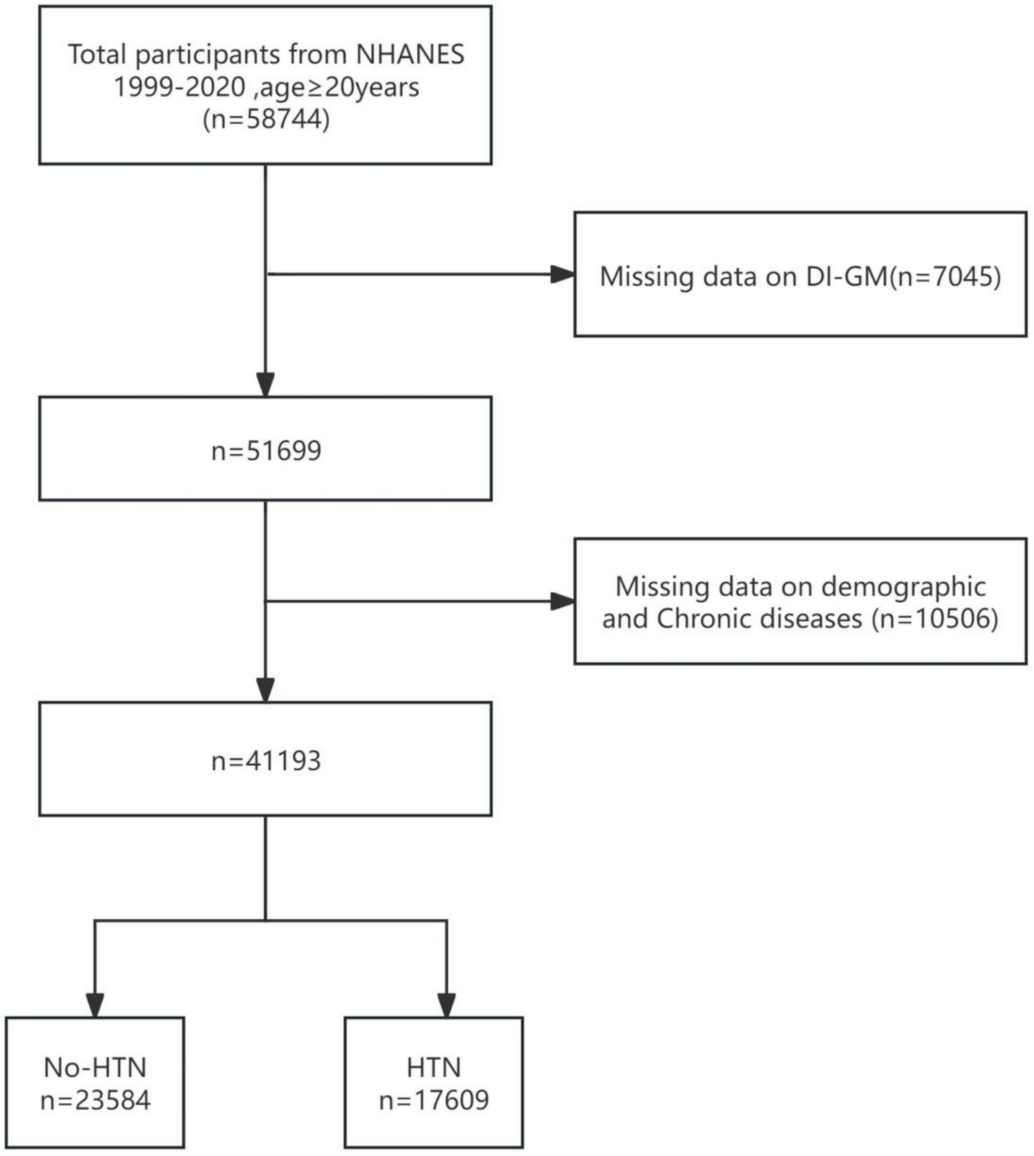
Flow chart of data filtering.

Data were extracted from the National Health and Nutrition Examination Survey (NHANES) database, which annually surveys approximately 5,000 individuals nationwide. NHANES conducted 11 cycles of surveys between 1999 and 2020. NHANES has received ethical approval from the CDC’s research ethics review board [NHANES 1999–2004: Protocol #98–12; NHANES 2005–2010; Protocol #2005–06; NHANES 2011–2020: Protocol #2011–17, #2018–01 (Effective beginning October 26, 2017)]. NHANES ensures participant rights protection through informed written consent. For further details regarding the ethical review and consent procedures of NHANES, please refer to https://www.cdc.gov/nchs/nhanes/. The inclusion criteria for our study were as follows: adults aged 20 years or older with complete data on hypertension and the Dietary Intake Index for Gut Microbiota (DI-GM) (see Figure 1). A total of 41,193 individuals satisfied these criteria and were included in the analysis.

### Dietary index for gut microbiota

In this study, the scoring system developed by Kase et al. was utilized to calculate the Dietary Index for Gut Microbiota (DI-GM). This index is based on 14 food groups or nutrients that serve as components of DI-GM. Specifically, fermented dairy products, chickpeas, soybeans, whole grains, fiber, cranberries, avocados, broccoli, coffee, and green tea are classified as beneficial components, whereas red meat, processed meat, refined grains, and high-fat diets (≥40% energy derived from fat) are categorized as detrimental components. The DI-GM scores were calculated using the 24-h dietary recall data collected during the National Health and Nutrition Examination Survey (NHANES) in the United States between 1999 and 2020. For beneficial foods, a score of one is assigned if their intake reaches or exceeds the gender-specific median (the gender-specific median of the NHANES); otherwise, the score is 0. Conversely, for detrimental foods, a score of 0 is assigned if their intake reaches or exceeds the gender-specific median (or if fat intake constitutes ≥40% of total energy); otherwise, the score is one. The total DI-GM score ranges from 0 to 14 points, with beneficial foods contributing up to 10 points (Beneficial DI-GM, BGMS) and detrimental foods contributing up to four points (Detrimental DI-GM, UGMS) (8).

### Hypertension

According to the hypertension definition established by NHANES (National Health and Nutrition Examination Survey in the United States), hypertension is defined as: a self-reported physician diagnosis of hypertension, a systolic blood pressure (SBP) of ≥140 mmHg, a diastolic blood pressure (DBP) of ≥90 mmHg, or current use of antihypertensive medication (15).

### Covariates

Age and body mass index (BMI) are regarded as continuous variables. The gender of the participants is divided into two groups: male and female. Racial/ethnic classification includes non-Hispanic White, non-Hispanic Black, Mexican Americans, other Hispanics, and others. Marital status is classified into two categories: married or living with a partner, and unmarried, widowed, divorced or separated. Educational background is classified based on the “Adult Education Attainment Survey Questionnaire” for people aged 20 and above, covering the following categories: not having completed 9 years of compulsory education, not obtaining a diploma from nine to 12 years of education, graduating from high school or having equivalent qualifications, some university education or associate degree, graduating from university or higher education. Poverty income ratio is classified into low income (<1.30), middle income (1.30–3.49), and high income (≥3.50). Smoking status is recorded as never smoking, former smoker and current smoker. Drinking situation is classified as never drinking, former drinker and current drinker. The assessed health status includes cardiovascular diseases (CVD) (present or absent), diabetes (present or absent), and hyperlipidemia (present or absent). Furthermore, the diagnosis of cardiovascular diseases (CVD) was based on self-reported physician diagnoses obtained through individual interviews using standardized medical condition questionnaires. Participants were asked, “Has a doctor or other health professional ever told you that you have congestive heart failure, coronary heart disease, myocardial infarction, or stroke?” An affirmative response to any of these conditions was considered as an indication of CVD. Hyperlipidemia was diagnosed if any of the following criteria were met: (1) current use of lipid-lowering medications; (2) elevated triglyceride levels (≥150 mg/dL); or (3) high cholesterol levels [total cholesterol ≥200 mg/dL, low-density lipoprotein cholesterol (LDL-C) ≥ 130 mg/dL, or high-density lipoprotein cholesterol (HDL-C) < 40 mg/dL]. Diabetes diagnosis was determined based on the following criteria: (1) self-reported physician diagnosis; (2) glycated hemoglobin (HbA1c) level >6.5%; (3) fasting blood glucose ≥7.0 mmol/L; (4) random or 2-h post-load glucose level during an oral glucose tolerance test ≥11.1 mmol/L; or (5) current use of antidiabetic medications or insulin. For more detailed information on the measurement of covariates, please visit the NHANES website.^1^

### Statistical analysis

Given the inclusion of hematological variables in our study, Mobile Examination Center (MEC) weights were applied. Specifically, for the periods 1999–2000 and 2001–2002, the weight calculation formula was wtmec4yr × 2 ÷ (11.625); for 2003–2018, it was wtmec2yr ÷ (11.625); and for 2017–2020, the formula was WTMECPRP × 1.625 ÷ (11.625) (16).

Respondents were categorized into two groups based on their hypertension status: the non-hypertension group (No-HTN) and the hypertension group (HTN). For continuous variables, data were described using weighted means (mean ± standard error), and differences between groups were assessed using the Wilcoxon rank-sum test tailored for complex survey designs. Categorical variables were presented as counts (n) and weighted percentages (%), with analyses conducted using the Rao-Scott chi-square test. Additionally, a weighted logistic regression model was constructed to examine the associations of DI-GM, BGMS, and UGMS with the risk of hypertension. Results were reported as odds ratios (ORs) with 95% confidence intervals (CIs). Specifically, Model 1 adjusted only for age; Model 2 further adjusted for gender, race/ethnicity, poverty-income ratio (PIR), marital status, and education level; Model 3 extended Model 2 by incorporating additional covariates, including smoking status, alcohol consumption, body mass index (BMI), cardiovascular diseases (CVD), hyperlipidemia, and diabetes. Based on the compositional analysis of BGMS and UGMS, no significant multicollinearity was observed between the two variables. In Model 3, when analyzing BGMS (or UGMS), UGMS (or BGMS) was included as an additional covariate to account for potential confounding. All regression analyses incorporated survey weights, and continuous covariates with non-normal distributions were transformed using weighted quartile-based methods. To assess the robustness of the observed associations, pre-specified weighted subgroup analyses were performed, stratified by age, gender, BMI, and diabetes status, with interactions between subgroups evaluated using likelihood ratio tests. Furthermore, subgroup analyses based on UGMS were conducted for BGMS. Nonlinear relationships between DI-GM, BGMS, and UGMS with hypertension risk were explored using restricted cubic splines (RCS) with power constraints. All statistical analyses were conducted using R statistical software (version 4.2.2,^2^ R Foundation) and the WinStat statistical analysis platform (version 2.1, Beijing, China).

## Results

### Baseline characteristics

This study included a total of 58,744 participants aged 20 years or older from the National Health and Nutrition Examination Survey (NHANES) database, with data spanning the period from 1999 to 2020. Among these, 7,045 participants were excluded due to missing DI-GM (diet and metabolism-related data). A total of 10,506 participants were excluded due to incomplete demographic and chronic disease-related information, which included 9,032 participants excluded for missing data on age, race, marital status, education level, poverty income ratio (PIR), smoking, alcohol consumption, or body mass index (BMI), as well as an additional 1,204 participants excluded due to missing data on specific chronic conditions, including hypertension, cardiovascular disease, hyperlipidemia, and diabetes. Ultimately, 41,193 participants who met the inclusion criteria were enrolled in the study. Figure 1 presents the participant selection flowchart. Table 1 summarizes the baseline characteristics of the study population. Statistical analyses identified significant differences across several variables, including age, race/ethnicity, poverty-income ratio (PIR), educational attainment, smoking status, alcohol consumption, body mass index (BMI), and the presence of various chronic conditions (e.g., cardiovascular disease, hyperlipidemia, and diabetes).

**Table 1:** The weighted fundamental clinical characteristics of participants in the hypertension and non-hypertension groups.

Characteristic	Overall, *N* = 156,805,661 *n* = 41,193	No-HTN, *N* = 98,462,249 *n* = 23,584	HTN, *N* = 58,343,411 *N* = 17,609	*p*
Age, mean (SE),	47.00 (0.20)	41.28 (0.19)	56.64 (0.22)	<0.001
Sex, n (%)				0.29
Male	20,688 (49.25%)	11,851 (49.01%)	8,837 (49.65%)	
Female	20,505 (50.75%)	11,733 (50.99%)	8,772 (50.35%)	
Race, n (%)				<0.001
Non-Hispanic White	19,138 (70.62%)	10,782 (69.62%)	8,356 (72.31%)	
Non-Hispanic Black	8,628 (10.39%)	4,142 (8.99%)	4,486 (12.76%)	
Mexican American	6,761 (7.54%)	4,379 (8.91%)	2,382 (5.23%)	
Other Hispanic	3,243 (5.24%)	2,018 (5.89%)	1,225 (4.15%)	
Other race	3,423 (6.20%)	2,263 (6.59%)	1,160 (5.55%)	
Marry, n (%)				0.17
Married/living with partner	24,801 (64.13%)	14,398 (63.80%)	10,403 (64.70%)	
Never married/Other	16,392 (35.87%)	9,186 (36.20%)	7,206 (35.30%)	
PIR_group, n (%)				<0.001
Low income	12,258 (20.21%)	6,832 (19.90%)	5,426 (20.75%)	
Middle income	15,644 (35.58%)	8,735 (34.55%)	6,909 (37.31%)	
High income	13,291 (44.21%)	8,017 (45.55%)	5,274 (41.94%)	
Education, n (%)				<0.001
Less than 9th grade	4,321 (4.97%)	2,039 (4.13%)	2,282 (6.39%)	
9-11th Grade	5,822 (10.51%)	3,103 (9.65%)	2,719 (11.95%)	
High school grad/GED or equivalent	9,590 (24.02%)	5,219 (22.49%)	4,371 (26.61%)	
Some college or AA degree	12,133 (31.50%)	7,177 (31.59%)	4,956 (31.34%)	
College graduate or above	9,327 (28.99%)	6,046 (32.13%)	3,281 (23.70%)	
Smoke, n (%)				<0.001
Never	22,044 (53.59%)	13,353 (56.22%)	8,691 (49.14%)	
Former	10,413 (25.23%)	4,814 (21.17%)	5,599 (32.09%)	
Now	8,736 (21.19%)	5,417 (22.62%)	3,319 (18.77%)	
Drink1, n(%)				<0.001
Never	5,552 (10.69%)	2,929 (9.97%)	2,623 (11.91%)	
Former	6,902 (13.56%)	2,982 (10.63%)	3,920 (18.52%)	
Current	28,739 (75.75%)	17,673 (79.41%)	11,066 (69.57%)	
BMI (kg.m^2^), mean (SE)	28.88 (0.06)	27.68 (0.07)	30.91 (0.08)	<0.001
CVD, n (%)				<0.001
No	36,602 (91.34%)	22,549 (96.40%)	14,053 (82.82%)	
Yes	4,591 (8.66%)	1,035 (3.60%)	3,556 (17.18%)	
Hyperlipidemia, n (%)				<0.001
No	12,441 (31.01%)	8,844 (37.89%)	3,597 (19.40%)	
Yes	28,752 (68.99%)	14,740 (62.11%)	14,012 (80.60%)	
DM, n (%)				<0.001
No	34,104 (87.28%)	21,586 (93.92%)	12,518 (76.07%)	
Yes	7,089 (12.72%)	1,998 (6.08%)	5,091 (23.93%)	
DI-GM, mean (SE)	4.53 (0.02)	4.53 (0.02)	4.54 (0.02)	0.39
BGMS, mean (SE)	2.22 (0.01)	2.22 (0.02)	2.22 (0.02)	0.72
UGMS, mean (SE)	2.31 (0.01)	2.30 (0.01)	2.32 (0.01)	0.13

N represents the weighted count to reflect the population distribution, while n represents the unweighted actual sample size count. BGMS, beneficial gut microbiota score; BMI, body mass index; CVD, cardiovascular disease; DI-GM, dietary index for gut microbiota; DM, diabetes mellitus; HTN, hypertension; PIR, poverty income ratio; SD, standard deviation; UGMS, unfavorable gut microbiota score.

### The relationship between DI-GM, BGMS, and UGMS and the risk of hypertension

To evaluate the independent association between DI-GM and its subgroups (BGMS and UGMS) with the risk of hypertension, we constructed a multivariable weighted logistic regression model to examine their relationships (see Table 2). Table 2 summarizes the results of the multivariable weighted regression analyses for DI-GM, BGMS, and UGMS in relation to hypertension. To ensure robustness, we sequentially adjusted for potential confounding factors using three progressively adjusted models. Specifically, Model 1 included adjustment for age only; Model 2 further adjusted for gender, race/ethnicity, poverty-income ratio (PIR), marital status, and education level; and Model 3 extended Model 2 by incorporating additional covariates, including smoking status, alcohol consumption, body mass index (BMI), and comorbidities such as cardiovascular disease, hyperlipidemia, and diabetes. Additionally, when analyzing the association between BGMS (or UGMS) and hypertension risk, UGMS (or BGMS) was included as a covariate to account for potential confounding. The results indicated that, after adjusting for all potential confounders, both DI-GM and BGMS levels were significantly and inversely associated with the risk of hypertension. Specifically, each one-unit increase in DI-GM was associated with a 4% reduction in hypertension risk (OR = 0.96, 95% CI: 0.94–0.98, *p* < 0.001), while each one-unit increase in BGMS was linked to a 5% decrease in hypertension risk (OR = 0.95, 95% CI: 0.92–0.97, *p* < 0.001). Notably, the protective effect of BGMS appeared slightly stronger than that of DI-GM, as reflected by the lower odds ratio. In contrast, no statistically significant association was found between UGMS levels and the risk of hypertension (*p* = 0.249).

**Table 2:** Relationship between DI-GM and the risk of hypertension.

Characteristics	Crude model		Model 1		Model 2		Model 3	
	OR (95% CI)	*P*-value	OR (95% CI)	*P*-value	OR (95% CI)	*P*-value	OR (95% CI)	*P*-value
DI-GM	1 (0.98, 1.02)	0.689	0.89 (0.87, 0.90)	<0.001	0.92 (0.90, 0.94)	<0.001	0.96 (0.94, 0.98)	<0.001
BGMS	0.99 (0.97, 1.02)	0.587	0.87 (0.85, 0.89)	<0.001	0.93 (0.91, 0.95)	<0.001	0.95 (0.92, 0.97)	<0.001
UGMS	1.02 (0.99, 1.05)	0.204	0.93 (0.91, 0.96)	<0.001	0.94 (0.91, 0.97)	<0.001	0.98 (0.95, 1.01)	0.249

DI-GM, Dietary Index of Gut Microbiota; OR, Odds Ratio (OR <1 indicates protective effect); CI, Confidence Interval; PIR, Poverty Income Ratio. Adjusted variables: Model 1 included age. Model 2 adjusted for age, gender, race, PIR, marital status, and education. Model 3 extended Model 2 by incorporating smoking status, alcohol consumption, body mass index (BMI), cardiovascular disease, hyperlipidemia, and diabetes. In Model 3, UGMS was included as a covariate when analyzing BGMS, and BGMS was included as a covariate when analyzing UGMS.

### Analysis of the dose–response relationship between DI-GM, BGMS, and UGMS and hypertension

**Figure 2:**
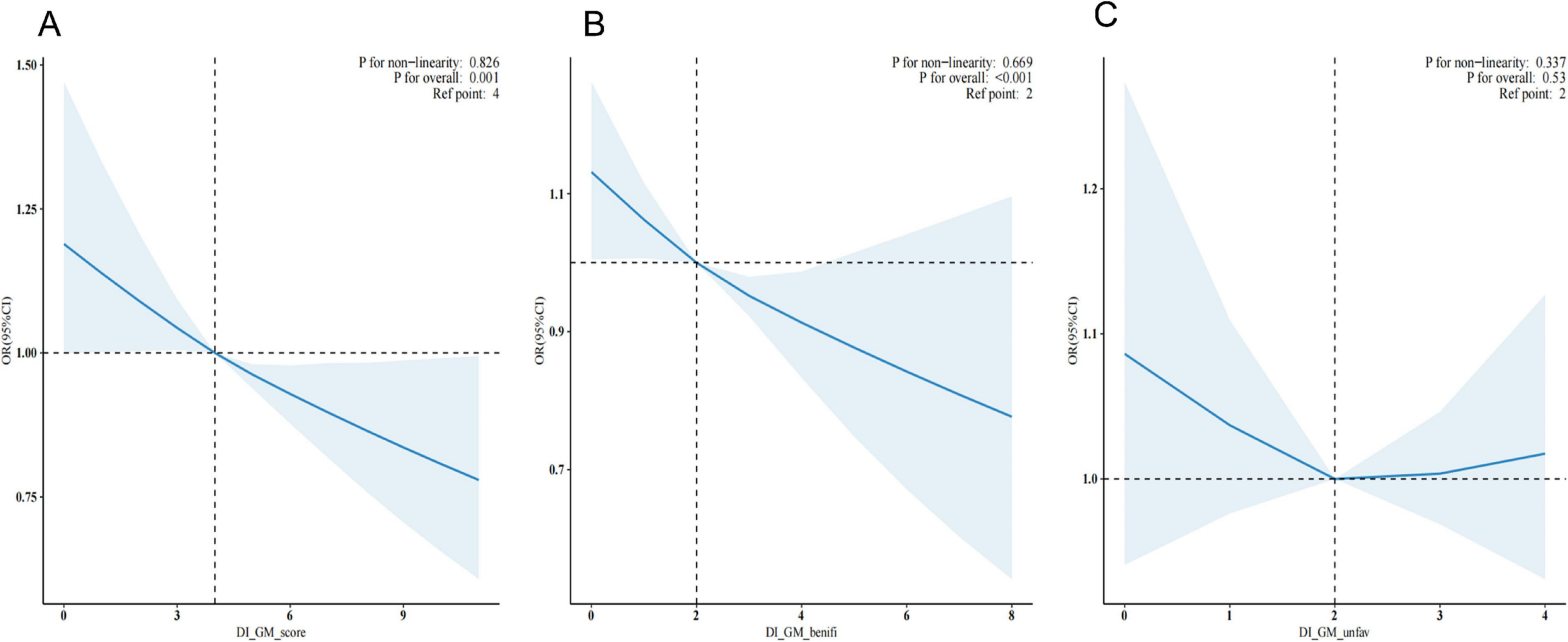
Restricted cubic spline plots depicting the additive interaction between DI-GM **(A)**, BGMS **(B)**, UGMS **(C)** and hypertension.

The dose–response relationship between DI-GM and its subgroups (BGMS and UGMS) and the risk of hypertension was systematically assessed using multivariable-adjusted restricted cubic spline (RCS) analysis (see Figure 2). The results demonstrated a significant linear association, with an inverse trend observed between DI-GM levels and the risk of hypertension (overall *p* = 0.001). Similarly, for the subgroup BGMS, a significant negative linear relationship was identified, indicating that as BGMS levels increased, the risk of hypertension significantly decreased (overall *p* < 0.001). In contrast, no significant dose–response relationship was observed between UGMS levels and the risk of hypertension (overall *p* > 0.05).

### Subgroup analysis of DI-GM, BGMS, and the risk of hypertension

**Figure 3:**
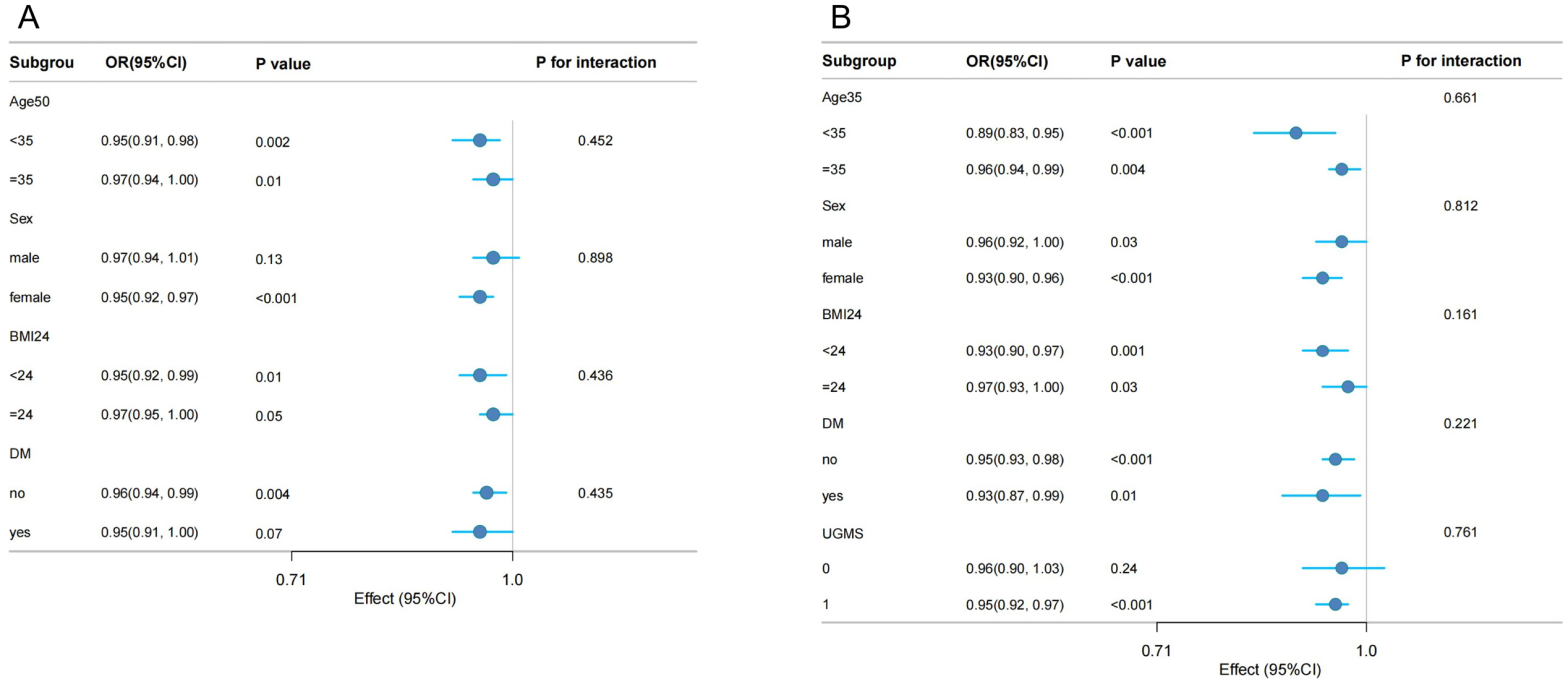
Forest plot of weighted subgroup analysis examining the association between DI-GM **(A)**, BGMS **(B)**, and hypertension.

Subgroup analyses were performed to evaluate whether the associations of DI-GM and BGMS with hypertension were modified by specific factors (see Figure 3). After adjusting for potential confounding variables, stratified analyses were conducted across age groups (<35 years and ≥35 years), gender, BMI categories (<24 and ≥24), and diabetes status (present or absent). The results indicated that the protective effects of DI-GM and BGMS remained consistent across all subgroups, with no statistically significant interactions observed (all interaction *p* > 0.05). Additionally, in the subgroup analysis for BGMS, further stratification by UGMS was included, with UGMS categorized into two groups based on its score (≤1 and >1). The findings indicated that in the subgroup with a UGMS score of ≤1, the association between BGMS and hypertension risk was not statistically significant (*p* > 0.05).

## Discussion

This study, utilizing data from the National Health and Nutrition Examination Survey (NHANES) conducted in the United States between 1999 and 2020, employed a cross-sectional research design to systematically evaluate the associations of the Dietary Index for Gut Microbiota (DI-GM), its beneficial component (Beneficial Gut Microbiota Score, BGMS), and its unfavorable component (Unfavorable Gut Microbiota Score, UGMS) with the risk of hypertension. After adjusting for demographic characteristics (age, gender, race/ethnicity), socioeconomic factors (poverty-income ratio, marital status, education level), lifestyle behaviors (smoking, alcohol consumption), and metabolic-related indicators [body mass index (BMI), history of cardiovascular disease, hyperlipidemia, and diabetes mellitus] using a multivariable logistic regression model, it was found that each one-unit increase in the DI-GM score was associated with a significant 4% reduction in the risk of hypertension (OR = 0.96, 95% CI: 0.94–0.98, *p* < 0.001). Similarly, each one-unit increase in the BGMS score was associated with a 5% decrease in the risk of hypertension (OR = 0.95, 95% CI: 0.92–0.97, *p* < 0.001). These findings suggest that both DI-GM and BGMS exert clear protective effects against hypertension, with BGMS, as the core component of DI-GM, demonstrating a stronger independent protective effect. In contrast, no statistically significant association was observed between UGMS and the risk of hypertension (*p* > 0.05). Further subgroup analysis revealed that among individuals with a UGMS score ≤1, the protective effect of BGMS was attenuated by the influence of the UGMS dietary pattern, resulting in a relatively higher risk of hypertension. These results suggest that even when adhering to the BGMS dietary pattern, failure to adequately control the intake of harmful components—such as red meat, processed meat, refined grains, and high-fat diets (with a fat energy ratio ≥40%)—may still contribute to an elevated risk of hypertension. The results of this study not only confirm the value of the overall DI-GM score and the BGMS component in hypertension prevention but also highlight the complex interplay between dietary patterns and gut microbiota, providing new insights for precision nutritional interventions.

To the best of our knowledge, this study represents the first systematic investigation into the association between the dietary index for gut microbiota (DI-GM) and the risk of hypertension. Current evidence suggests that the pathophysiology of hypertension involves intricate interactions between genetic predispositions and environmental influences. However, genome-wide association studies (GWAS) indicate that genetic factors account for less than 5% of the variance in hypertension incidence (17). Conversely, modifiable lifestyle factors exert a more pronounced influence on blood pressure regulation. For example, changes in body mass index (BMI) and sodium intake can lead to approximate fluctuations of 5 mmHg in blood pressure levels (18). In terms of dietary determinants, extensive prospective cohort studies have demonstrated that dietary patterns rich in fruits and vegetables, with restricted consumption of sweets and refined grains, and prioritizing healthy fats and proteins, are associated with a significant reduction in hypertension risk. Notably, both the Mediterranean diet and the DASH (Dietary Approaches to Stop Hypertension) diet, renowned for their antihypertensive properties, have been extensively validated (19–21). The Mediterranean diet confers cardiovascular protection by enhancing the production of short-chain fatty acids (SCFAs), with its core components—predominantly plant-based foods supplemented with olive oil, moderate amounts of poultry and fish, and limited red meat—proven to effectively mitigate hypertension risk (22). This study specifically highlights the potential utility of the DI-GM score, which exhibits a robust positive correlation with the classical Mediterranean Diet Score (MDS) (*r* = 0.42, *p* < 0.0001) (23). These findings suggest that the DI-GM score, grounded in gut microbiota characteristics, may serve as a novel quantitative metric and intervention target for dietary strategies aimed at preventing hypertension.

Extensive research has demonstrated that dietary nutrient intake exerts a significant regulatory influence on the structure and functional activity of trillions of microorganisms residing in the human gut (24–28). A prospective cohort study conducted in the Chinese population revealed that, compared to healthy controls, patients with hypertension exhibited a marked reduction in intestinal microbiota diversity (29). Of greater significance, animal experiments utilizing fecal microbiota transplantation technology have provided direct evidence of the causal relationship between the intestinal microbiota and hypertension: when the intestinal microbiota from spontaneously hypertensive rats (SHRSP) was transplanted into normotensive Wistar-Kyoto (WKY) rats, the recipient rats displayed a substantial increase in blood pressure (30). Current evidence highlights significant differences in the composition of intestinal microbiota between hypertensive patients and healthy individuals. These differences are primarily characterized by: (1) a reduced abundance of short-chain fatty acid (SCFA)-producing bacteria, such as Roseburia and Faecalibacterium; and (2) an increased relative abundance of Gram-negative bacteria (5). Mechanistically, SCFAs, as key metabolites derived from the fermentation of dietary fiber by gut microbiota, regulate blood pressure via the “gut-vascular axis.” Specifically, after absorption into the circulatory system, SCFAs activate host receptors such as Olfr78 and Gpr41, thereby modulating blood pressure homeostasis through the regulation of renin secretion and vascular tone (31, 32). Conversely, lipopolysaccharide (LPS), a major component of the cell wall of Gram-negative bacteria and a potent endotoxin, may contribute to the development of hypertension through multiple pathophysiological mechanisms, including systemic inflammation, activation of the sympathetic nervous system, and neuroinflammation induction (5). In this study, the DI-GM scoring system was developed based on rigorous scientific evidence, with its components selected for their regulatory effects on intestinal microbiota diversity, SCFA production capacity, and the abundance of specific beneficial bacterial taxa (8).

Aging is a major independent risk factor for the development of hypertension. Epidemiological data from the Framingham Heart Study in the United States indicate that individuals aged 55–65 have a lifetime risk of developing hypertension as high as 90%. Among those aged 65–89, 87% of men and 93% of women with hypertension exhibit isolated systolic hypertension (33). Baseline analyses reveal that without adjustment for confounding factors such as age, the associations between DI-GM, BGMS, and UGMS and hypertension do not achieve statistical significance. However, in the multivariate weighted logistic regression model (Model 1), after adjusting solely for age, DI-GM and BGMS demonstrate significant statistical associations with hypertension (*p* < 0.001). Further subgroup analyses show that regardless of whether groups are divided at the age boundary of 35 (<35 years vs. ≥35 years), DI-GM and BGMS exhibit significant protective effects against hypertension.

The significant findings of this study not only enhance our understanding of the interaction mechanism among diet, gut microbiota, and hypertension but also represent a critical breakthrough in translating basic research into clinical application. The quantitative assessment tool and well-defined intervention targets developed in this study provide a robust scientific foundation for transforming hypertension prevention and control models, facilitating a transition from traditional “disease treatment” approaches to more advanced “health maintenance” strategies.

However, this study also has several limitations that warrant consideration. First, the cross-sectional design of the National Health and Nutrition Examination Survey (NHANES) database precludes the establishment of a definitive causal relationship. Future prospective cohort studies are needed to validate the temporal associations observed. Second, the DI-GM index is constructed based on intake data for 14 specific dietary components; any missing data for these components results in sample exclusion, which may introduce potential selection bias during the screening process. Furthermore, despite rigorous adjustment for multiple known confounding factors, residual confounding effects and the influence of unmeasured factors (e.g., genetic background) cannot be entirely ruled out. Finally, the reliance on self-reported dietary intake data and covariate information may introduce issues such as recall bias.

## Conclusion

This study found a significant negative correlation between the dietary index of gut microbiota (DI-GM) and the risk of hypertension in adults over 20 years old. Among them, the protective effect of the dietary score of beneficial gut microbiota (BGMS) was more significant. These findings highlight the potential of diet intervention measures focusing on gut microbiota as a promising strategy for preventing hypertension.

## Data Availability

Publicly available datasets were analyzed in this study. This data can be found at: https://www.cdc.gov/nchs/nhanes/index.htm.

## Footnotes

^1^ https://www.cdc.gov/nchs/nhanes/index.htm

^2^ http://www.R-project.org
